# Uterine contractile effects of the aqueous and ethanol leaf extracts of *Newbouldia Laevis* (Bignoniaceae) *in vitro*

**DOI:** 10.4103/0250-474X.54274

**Published:** 2009

**Authors:** E. Bafor, U. Sanni

**Affiliations:** Department of Pharmacology and Toxicology, Faculty of Pharmacy, University of Benin, 300 001, Nigeria

**Keywords:** *Newbouldia laevis*, uterine contraction, spontaneous contraction, nonpregnant rats

## Abstract

Based on traditional reports, the aqueous and ethanol extracts of the leaves of *N. laevis* were tested on isolated uterine preparations of non-pregnant rats. The effects of increasing cumulative concentrations of the extracts on the amplitude and frequency of spontaneously contracting uterine tissues were tested. Direct effects of the extracts and acetylcholine on uterine smooth muscle were also tested in organ baths containing aerated physiological salt solution maintained at 37°. The EC_50_ and Emax were determined and analyzed using one way ANOVA with Dunnett's post hoc test. The extract significantly increased the frequency (*P*<0.05) of spontaneous contractions without significantly affecting the amplitude. The extracts and acetylcholine were observed to directly stimulate uterine contractions, however there were significant differences (*P*<0.05) in their EC_50_ and Emax. In conclusion, the leaves of *N. laevis* increase the frequency of spontaneously contracting tissues and directly stimulate uterine contractions which may account for the use of the leaf extract traditionally.

The use of plants to facilitate birth or to protect the young embryo appears to be a common practice among traditional healers. *Newbouldia laevis* is one of such plants and its leaves are used in Southeastern Nigeria to hasten parturition and to expel the placenta after delivery[1]. Agents that stimulate uterine contraction are classified as oxytocics[2] and are employed clinically for the induction and augmentation of labour as well as in the management of the third stage of labour[3]. Thus *N. laevis* as used by traditional healers falls under the category of oxytocics, however this classification remains to be proven. This study was hence undertaken to investigate the purported oxytocic activity of the leaves of *N. laevis*.

*N. laevis* is a medium sized angiosperm in the Bignoniaceae family. It is native to tropical Africa and grows to a height of about 10 m with a cauliferous habit. It is ever green, though its leaves turn somewhat dark purple during the cold seasons[4]. It is popularly known as the tree of life or fertility tree in Nigeria. Its local Nigerian names include *Akoko* (Yoruba), *Aduruku* (Hausa), *Kontor* (Tiv), *Ikhimi* (Bini), *Ogirisi* (Igbo) and *Ogiriki* (Urhobo).

Fresh leaves of *N. laevis* were collected in Benin City, Edo state of Nigeria between the months of March and April. The plant was identified at the Department of Botany and the Department of Pharmacognosy, University of Benin, Nigeria.

Diethylstilboestrol and acetylcholine (ACh) used in this study were obtained from Sigma (UK). The drugs were prepared fresh on the day of the experiment by dissolving in physiological salt solution (composition stated above) with the exception of diethylstilboestrol, which was constituted in ethanol obtained from Sigma (UK).

The leaves were cleaned, air-dried for 5 days and ground into a powder. A portion of the powder was macerated in distilled water for 48 h and a separate portion was macerated in ethanol for 24 h. The extract was decanted, filtered and concentrated in a vacuum evaporator (Buchi R110, Germany) at 60° and dried in an oven set at 40°. The aqueous sample gave a yield of 1.85% w/w and the ethanol sample gave a yield of 1.24% w/w.

Female Sprague-Dawley rats (160-180 g) were used. They were purchased from Ambrose Alli University Ekpoma and were housed locally at the Laboratory Animal Unit of the Department of Pharmacology and Toxicology, University of Benin, Nigeria. The animals were allowed an acclimitazation period of one month before their use for the experiment and were maintained under standard conditions and had free access to standard diet and water. They were handled according to standard guidelines for use of laboratory animals[5].

The animals were pre-treated with diethylstilboesterol (0.2 mg/kg, ip) 24 h prior to the commencement of the experiment. Oestrus was confirmed by microscopic observation of vaginal smears and macroscopic observation of the vulva. The rats were sacrificed under chloroform anesthesia. Uterine segments, 2 cm in length were rapidly dissected out and freed of adhering tissues. These were mounted in 40 ml organ baths containing physiological salt solution of the following composition in g/5 l: NaCl 45.0, NaHCO_3_ 2.5, D-glucose 2.5, KCl 2.1, and CaCl_2_.2H_2_O 1.32. The lower end of the tissue was attached to a tissue holder by means of silk suture and the upper end to a Ugo Basile isometric force-displacement transducer (model 82145) connected to a Ugo Basile unirecorder (model 7050). The solution was maintained at 37° and continuously aerated. The preparations were equilibrated for 45 min at resting tension of 0.75 g before the start of the experiment.

After equilibration, spontaneous control contractions (amplitude and frequency) were recorded during the first 10 min period and this was taken as 100%[6]. This was followed by subsequent 10 min exposure of the tissue to the extract at increasing cumulative concentrations[7]. The tissue was exposed to cumulative extract concentrations of 0.05×10^−2^ to 200×10^−2^ mg/ml and the responses observed. The maximum force displacement per contraction and the number of uterine contractions per min were used to calculate the average force and frequency of uterine contractions for 10 min intervals. The average force and number of contractions/min measured during the 10 min period immediately prior to exposure were used as the basal contraction force and frequency. The aqueous and ethanol extracts, 0.05×10^−2^ to 200×10^−2^ were added non-cumulatively to the organ baths containing uterine tissue with a contact time of 45 s and responses observed. Concentration response relationship to non-cumulative additions of acetylcholine (0.6×10^−4^ to 2.5×10^−4^ mg/ml)[8] to the organ baths containing uterine tissues was determined with a contact time of 30 s.

All values are expressed as mean±SEM (standard error of mean) and *n* represents the number of rats from which uterine segments were obtained. The EC_50_ (concentration which produced 50% of maximum response) and E_max_ (maximum achievable response) were computed for each concentration-response experiment. Comparisons were made using one-way ANOVA with Dunnett post hoc test. Statistical significance of *P*< 0.05 was used in all cases.

The ethanol extract had no significant effect on the amplitude of spontaneous rat uterine contractions but however significantly increased (*P*<0.05) the frequency (Table 1). The aqueous extract similarly produced no significant effect on the amplitude of spontaneous contractions but significantly increased (*P*<0.05) the frequency of spontaneous rat uterine contractions (Table 2). The aqueous and ethanol extracts of *N. laevis* stimulated uterine contractions in a concentration- dependent manner as shown in fig. 1. ACh stimulated uterine contractions in a concentration-dependent manner as shown in fig. 2. The EC_50_ of the aqueous extract of *N. laevis* was computed to be 0.42×10^−2^ mg/ml, the ethanol extract was 0.29×10^−2^ mg/ml while that of ACh was 39×10^−5^ mg/ml as shown in Table 3.

**TABLE 1:** EFFECT OF THE ETHANOL EXTRACT OF *N. LAEVIS* ON RHYTHMIC SPONTANEOUS UTERINE CONTRACTIONS

Concentration (×10^−2^ mg/ml)	% Mean amplitude±SEM	% Mean frequency±SEM
0 (baseline)	100	100
0.39	100	100
0.78	100	100
1.56	100	100
3.12	107.3±2.2	144±3.3*
6.25	116.9±4.7	203±2.9*
12.5	151.1±6.1	228±3.2*
25	153.8±6.2	226±2.1*
50	168.5±6.8	208±1.3*
100	163.2±6.6	181.7±2.2
200	171.5±7.0	168.7±2.1
400	181.9±7.5	157.3±2.3
800	139.5±5.7	73.2±6.9

The ethanol extract of *N. laevis* significantly increased (*P*<0.05) the frequency without significantly affecting amplitude of spontaneous contractions.

* *P*<0.05 compared to baseline; n= 5 rats

**TABLE 2:** EFFECT OF THE AQUEOUS EXTRACT OF *N. LAEVIS* ON RHYTHMIC SPONTANEOUS CONTRACTIONS

Concentration (×10^−2^ mg/ml)	% Mean amplitude±SEM	% Mean frequency±SEM
0 (baseline)	100	100
0.39	100	100
0.78	100	100
1.56	100	102±5.2
3.12	103.2±1.2l	153±3.5*
6.25	105.4±2.4	214±3.2*
12.5	101.2±5.4	225±6.4*
25	105.2±7.7	235±6.9*
50	105.6±5.9	211±5.6*
100	103.3±5.3	102.4±6.6
200	109.1±8.9	105.1±7.1
400	107.3±4.1	92.3±7.8
800	95.6±3.3	93.5±7.4

The aqueous extract of *N. laevis* significantly increased (p<0.05) the frequency without significantly affecting amplitude of spontaneous contractions.

* *p*<0.05 compared to baseline; n= 5 rats

**Fig. 1:**
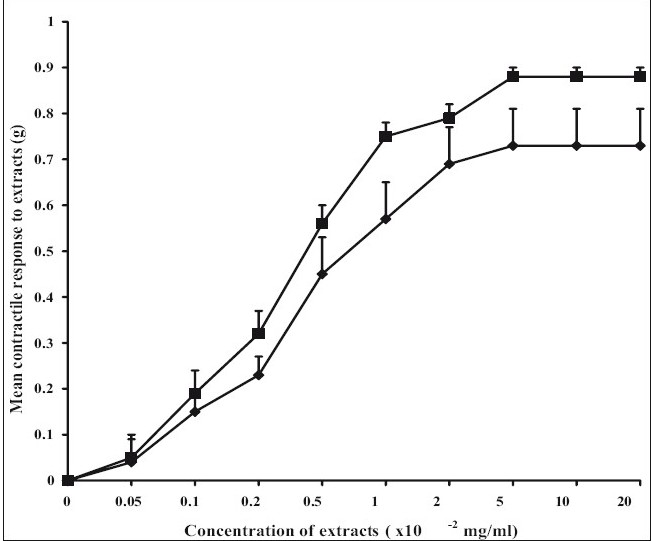
Concentration-response curves to the aqueous and ethanol extracts of *N. laevis* The aqueous (–◆–) and ethanol extract (–■–) of *N. laevis* apparently stimulate uterine activity. They stimulated the uterus equipotently. There was no significant difference in the E_max_ of both extracts.

**Fig. 2:**
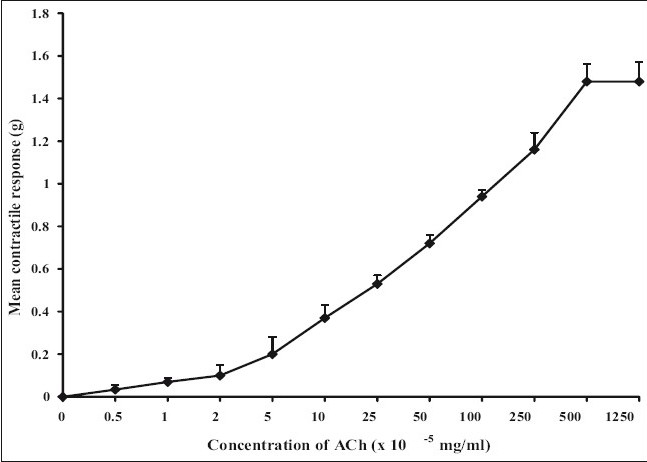
Concentration-response curve of ach ACh (–◆–) dose-dependently contracted isolated rat uterine preparations. The manner of ACh- induced contraction was compared to those of the extract. They both produced concentration-dependent contractions with no significant difference in the E_max_.

**TABLE 3:** CONCENTRATION OF EXTRACT PRODUCING 50% OF MAXIMUM RESPONSE

Drug/Extract	EC_50_ (mg/ml)
Aqueous extract	0.0042±0.00017*
Ethanol extract	0.0029±0.0002*
Acetylcholine	0.00039±0.00002

The values represent the means±SEM for 5 rats per group. Statistically significant differences between groups were measured using one-way ANOVA with Tukey-Kramer Multiple Comparison Test.

* P<0.05 compared to ethanol extract as well as compared to ACh.

The increase in frequency by the extracts (aqueous and ethanol) suggests that the extracts may increase the open state probability of voltage-dependent calcium channels, allowing an influx of extracellular calcium and enhancing contractions or the extracts may interfere with voltage-gated potassium channels which has been proposed as a major contributing factor to basal myometrial contractility[9]. The extracts may also promote directly or indirectly, production of prostaglandins *in situ*. Local prostaglandin production has been suggested to contribute to the maintenance of smooth muscle activity[10]. However, the increase in frequency by the extracts may also have resulted from activation of receptor-operated calcium channels. It was however observed that concentrations of extracts that produced an increase in frequency of contractions appeared to have no significant effect on the amplitude of spontaneously contracting uterine tissues. This may have occurred because the uteri used for this experiment were oestrogen dominated which places the uterus in a state of increased sensitivity[11], thus baseline uterine contractions may have been at their peak and agonists which would have otherwise increased the force of contraction would appear to have no effect on force rather their effect would only be observed in the frequency of contraction. A second probable reason for this seeming lack of effect on amplitude of spontaneous contractions might be that the extracts had no direct effect on the endogenous pacemaker cells, which resides in uterine tissues[12]. Thus, the extracts will have no effect on gap junction assembly and will not enhance or inhibit cellular communication, culminating in stable or unaffected amplitude of uterine contraction.

The aqueous and ethanol extracts of *N. laevis* have been shown to directly stimulate uterine contractility in a concentration dependent manner (fig. 1). The lack of significant difference between the extracts (Table 3) suggests that the solvent ethanol was just as effective in the extraction of the active contractile constituent of the crude plant extract compared with water (the aqueous solvent). ACh, a known muscarinic agonist also produced a concentration related increase in uterine contraction as shown in the results. This agonist effect is due to its direct interaction with specific muscarinic receptors in the uterine smooth muscle.

All muscarinic receptors appear to be of the G-protein family with seven segments arranged in serpentine fashion across the membrane. An important result of muscarinic agonist binding is activation of the inositol triphosphate (IP_3_) and diacylglycerol (DAG) cascade. Some evidence implicates DAG in the opening of the smooth muscle calcium channels. IP_3_ evokes release of calcium from the endoplasmic and sarcoplasmic reticulum. Muscarinic agonists also increase cellular concentration of cyclic GMP. Activation of muscarinic receptors also increases potassium flux across cell membrane. An effect mediated by direct binding of an activated G-protein to the channels[13].

The extracts and ACh contracted the uterus similarly as observed by the concentration-dependent increase in contractility as well as in the lack of significance in the E_max_ produced. These findings indicate that the aqueous and ethanol extracts of the leaves of *N. laevis* increase the frequency of spontaneous uterine contractions and directly stimulate contraction of the uterus. This effect is similar to the contraction produced by ACh, a muscarinic agonist. The contractile effect of the extract may be the reason for its use by traditional healers in Southern Nigeria to facilitate and augment labour.
